# GWAS on early sexual maturation across freshwater and seawater environments in domesticated Lochy strain of Atlantic salmon

**DOI:** 10.1186/s12711-025-01026-5

**Published:** 2026-01-03

**Authors:** Patricia Rivera, M. Angélica Rueda-Calderón, Nicol Delgado, María Eugenia López, Anti Vasemägi, Carlos Soto, Alfonso Romero, José Gallardo-Matus

**Affiliations:** 1 Laboratorio de genética y genómica aplicada, Pontificia Universidad Católica de Valparaíso, Escuela de Ciencias del Mar, Valparaiso, Chile; 2 Institute of Freshwater Research, Department of Aquatic Resources (SLU Aqua), Swedish University of Agricultural Sciences, Stångholmsvägen 2, Drottningholm, Uppsala, 17893 Sweden; 3 Chair of Aquaculture, Institute of Veterinary Medicine and Animal Sciences, Estonian University of Life Sciences, Kreutzwaldi 46, Tartu, 51006 Estonia; 4 Genetics, Reproduction and R&D Area, Salmones Camanchaca, Puerto Montt, Chile

## Abstract

**Background:**

Early sexual maturation is a challenging obstacle to overcome in Atlantic salmon farming. This trait primarily affects males and occurs in both freshwater fish farms and sea culture cages during the fattening phase. Current strategies for preventing early maturation include a combination of genetic selection and management practices. However, the genetic architecture of early maturation appears to vary across populations, strains and environments. Our study aimed to elucidate the genetic architecture of early maturation in the Lochy strain of Atlantic salmon using genome-wide SNP panels. This European-origin strain grows rapidly but is prone to high rates of precocious male maturation if not properly managed.

**Results:**

We report two genome-wide association (GWAS) results focusing on males of the Lochy strain of Atlantic salmon. The first included seawater-cultured fish (Group-SA: 714 males, 80 precocious and 634 immature) with an artificial continuous light photoperiod, while the second included freshwater-cultured fish (Group-FN: 707 males, 333 precocious and 374 immature) with a natural photoperiod. Group-SA was genotyped using a custom 46,115-SNP Illumina microarray, whereas Group-FN employed a custom 62,044-SNP Thermo microarray. Genomic heritability of early maturation in males was consistently high across models—ranging from 0.62–0.79 in seawater and from 0.54–0.62 in freshwater. In Group-SA, one significant SNP associated with early sexual maturation were identified on chromosome Ssa25. In Group-FN, sixty significant SNPs associated with early sexual maturation were identified on chromosomes Ssa5, Ssa7, and Ssa25. The genetic variance explained by these SNPs ranged from 16.1–53.7%, while the proportion of phenotypic variance explained varied from 8.7% to 29.1%. The identified candidate genes included *chmp2b* and *vgll3*, both previously reported in other domesticated European-origin populations, suggesting some degree of convergence.

**Conclusions:**

The SNPs associated with early maturation are promising candidates for application in breeding programs in the Lochy strain aimed at implementing improved control strategies against early maturation in both freshwater and sea environments.

**Supplementary Information:**

The online version contains supplementary material available at 10.1186/s12711-025-01026-5.

## Background

The presence of early-maturing individuals within salmon aquaculture settings has negative effects on productivity and animal welfare [1–4]. Early maturation is a heritable trait in wild Atlantic salmon populations [5] and plays a significant role in shaping the life history strategies of this species [6]. However, in farmed environments, this phenomenon poses a significant challenge for salmon farmers and has proven to be a persistent phenomenon that is difficult to eradicate [7, 8]. Although both males and females show early maturation, it is more prevalent in males [6]. From a productivity perspective, early maturing fish experience growth cessation, resulting in decreased yield at harvest [4, 9]. Early maturing fish also undergo external changes, such as the development of secondary sexual characteristics, darkening of skin pigmentation, and the formation of a distinctive hooked jaw in males [10, 11]. Furthermore, early maturation alters the composition of the meat by diverting energy from growth to reproduction, resulting in a decrease in fillet color [4]. This fading of fillet coloration is attributed to the mobilization of astaxanthin from muscle to skin [12]. In terms of animal welfare, the presence of precocious mature fish is associated with decreased immune defense [13], an increased risk of disease, and social stress [4]. Precocious mature fish are usually sacrificed before harvest, and the registration of this procedure is mandatory in some countries, such as Chile, revealing that thousands of fish reach precocious maturity every year [Additional file 1, Figure S1].

To mitigate the negative impacts of early maturation, aquaculture sector employs a range of production management techniques, including photoperiod manipulation and strategic smolt timing. Photoperiod, which is the primary signal for seasonal modulation of sexual maturation in salmonids [14, 15], is artificially modified as the main strategy for controlling early maturation in Atlantic salmon farming [7, 16]. For instance, studies have demonstrated that the use of continuous artificial light under halogen lamps reduced the incidence of precociously mature males from 74–16% compared to that of fish reared under natural lighting alone [14]. More recent research [15] utilizing LED lights confirmed that high-intensity light superimposed on natural light in cage culture promotes growth and reduces the occurrence of sexual maturation. Currently, the adoption of LED lights as a standard practice has become widespread in the aquaculture industry to enhance growth and minimize maturation [17]. Despite this advances, early maturity still persists in domesticated Atlantic salmon populations, underscoring the potential genetic component associated with this trait [18].

Moderate heritability of sexual maturation in Atlantic salmon has been recognized for nearly four decades [4], leading to the proposal and implementation of artificial selection schemes to reduce the incidence of early maturation in farmed populations [19, 20]. Recently, with the advent of GWAS, it has become possible to unravel the genetic architecture underlying this trait. Studies have shown that in various populations of Atlantic salmon, the genetic architecture associated with age at maturity involves key loci. In freshwater, Mohamed et al. [9] identified the strongest associations with maturation with SNPs on Ssa10 and 11, and linked to genes like *picalm* and *magi2*, both of which are implicated in reproductive and developmental processes. For seawater maturation, *vgll3* on chromosome Ssa25 has been identified as one of the key genes [21, 22]. Barson et al. [23] found that the *vgll3* locus explains 39% of the phenotypic variation in sea age. Additionally, other significant loci associated with sea age have been identified, including gene *six6* on chromosome Ssa09 [24]**,** which has also been linked to seasonal river return timing in Atlantic salmon accounting for 24% of the phenotypic variation [25].

Furthermore, the genetic architecture of early maturation not only varies between freshwater and sea environments but also appears to be population specific. For example, [10] did not find a relationship between the *vgll3* gene and age at maturity in females of the domesticated Mowi strain of Atlantic salmon, contrary to observations in wild populations. Similarly, [3] found no association between age at maturity and the *vgll3* gene in a domesticated North American population. The differences in genomic regions associated with age at maturity in Atlantic salmon across studies may be attributed to several factors. Smaller sample sizes can reduce the statistical power to detect associations. Additionally, in certain populations, genetic variants may be near fixation or already fixed, limiting the ability to identify associations. Other contributing factors include rearing environmental conditions, which can lead to contrasting phenotypic expressions [2]. These divergences in the genetic architecture of age at sexual maturity are not limited to Atlantic salmon, as studies have also identified that the *vgll3* and *six6* genes contribute differently to sexual maturation in other species [26, 27]. These circumstances highlight the need to characterize the genetic architecture of early maturation across divergent populations more comprehensively.

The Lochy strain originates from the Lochy River in the Highland region on the west coast of Scotland [28] and was introduced to Chile in the 1990s, after which it became domesticated and genetically isolated from other strains. The Lochy strain is characterized by having high feed consumption rates, growing quickly during the fattening stage, and having a production cycle of three years, which is shorter compared to other Atlantic salmon strains, whose production cycles typically last four years [29]. Despite its rapid growth, the Lochy strain is susceptible to high rates of early sexual maturation in both freshwater and sea environments. To manage this phenomenon in the Lochy strain, producers employ a combination of selective breeding—removing early-maturing males and females—and strategic management practices, such as implementing a 24-h light photoperiod during sea cage production and carefully timing smolt stocking to avoid two winters at sea.

The objective of this study was to explore the genetic architecture underlying early sexual maturation in male Atlantic salmon of the Lochy strain. In this work, the investigation of the genetic architecture of early sexual maturation using genome-wide association approach was conducted on two distinct groups of the Lochy strain, the first cultured in seawater with an artificial photoperiod (Group-SA) and the other reared in freshwater with a natural photoperiod (Group-FN).

## Methods

### Study groups and culture conditions

Our study included male fish from the Lochy strain, which were obtained from the Atlantic salmon genetic improvement program of Salmones Camanchaca, Chile. We analyzed two distinct experimental groups: Group-SA and Group-FN [Additional File 1, Figure S2]. Group-SA comprised 714 males, 80 precocious and 634 immature (Table 1) sampled in 2020 from two sea culture centers, Contao and Manihueico (Los Lagos region, Chile). Briefly, the fish in Group-SA had no known pedigree and were reared under a continuous artificial light photoperiod from the smolt stage (mean stocking weights: Contao = 147 g; Manihueico = 167 g) until harvest. The photoperiod consisted of 24 h of light (24L:0N) provided by four 600-W bulbs. In each center, there was a single-sex cage designated for rearing male fish of the Lochy strain, with an initial stocking of 75,000 smolts. For the Group-SA, male smolts from the Contao center were stocked in April 2019 (late autumn) with 5395-degree days, while those from the Manihueico center were stocked in June 2019 (early winter) with 5268-degree days. Harvesting from both sea culture centers took place in winter 2020 (Contao: July; Manihueico: August), after which fish were transported to the primary processing plant in Calbubo (Los Lagos region, Chile). Here, selective sampling (phenotyping) was performed, classifying males as precocious mature or immature based on secondary sexual characteristics, including hooked jaw, body size, and skin coloration. Due to photoperiod control, a low prevalence of mature individuals was observed. A summary of the productive parameters during phenotyping for the fish sampled from both culture centers (precocious and immature) is provided in Table 1. Briefly, the precocious mature fish were smaller and lighter [Additional file 1, Figure S3–S4], and their gonads were heavier than those of the immature fish, resulting in a significantly greater gonadosomatic index (GSI) in the precocious mature fish than in the immature fish [Additional File 1, Figure S5]. Group-FN consisted of 707 males, including 333 precocious and 374 immature individuals (Table 1), sampled in 2021 from the Freshwater Polcura Fish Farm (Bio Bío Region, Chile). These fish have a known pedigree and belong to 142 full-sib families, with the number of full siblings ranging from 1–18 (mean: 5; mode: 3). The FN group was reared under freshwater conditions and a natural photoperiod throughout their entire life cycle. The biological samples for the FN-Group were obtained through two sampling events from a cohort of 4767 broodstock, including 2119 males. The first selection sampling took place in June of 2021, in early winter when the fish were 24 months (7358-degree days). At this time, 333 individuals exhibiting early sexual maturation within the culture environment were identified and selected. In December 2021, when the remaining group of animals were 30 months, a second sampling of adult fish was conducted, during which samples of immature males were collected. Individuals displaying deformities, low body condition, or ocular damage were excluded, resulting in 374 non-matured salmon for the study. In Group-FN, WFE weight was not measured, as this metric is not commonly used in freshwater productions. Moreover, as it was not possible to obtain morphometric data for all individuals in Group-FN in the field, morphometric variables, including weight, length, gonad weight and gonadosomatic index (GSI), were measured in a random subsample of 20 precocious mature and 20 immature fish in June of 2021 (Table 1) [Additional File 1, Figure S5]. A comparative image of external physiognomy and gonadal weights between precocious mature and immature fish in this group is provided in [Additional file 1, Figure S6].

**Table 1:** Descriptive statistics for morphological and maturation traits classified by fish farm and group (SA: seawater; FN: freshwater)

Fish farm/Group	Maturity	Fishes for GWAS	Length (cm)	Weight (g)	Gonad weight (g)	GSI (%)
Contao group-SA	Immature	327	79.7 ± 4.8	6143.3 ± 1254.7	5.4 ± 3.5	0.08 ± 0.1
	Precocious mature	6	77.7 ± 5.0	4723.8 ± 1421.8	256.7 ± 170.6	5.1 ± 3.6
Manihueico group-SA	Immature	307	77.9 ± 4.8	6323.0 ± 1258.6	5.3 ± 3.8	0.08 ± 0.1
	Precocious mature	74	74.6 ± 5.3	4940.5 ± 997.0	222.6 ± 73.1	4.2 ± 1.3
Polcura group-FN	Immature	374	57.2 ± 1.8	2533.6 ± 276.5	1.2 ± 0.6	0.05 ± 0.0
	Precocious mature	333	56.3 ± 2.6	2178.9 ± 305.2	127.1 ± 41.2	5.9 ± 2.1

In the context of Group-FN, the table provides a summary of data for a subsample comprising 20 mature and 20 immature fish due to the lack of this information for the entire population used in the GWAS. Furthermore, for Group-SA, the table summarizes the wet fish equivalent (WFE) weight, whereas for Group-FN, it summarizes the live weight of the fish

### Genotyping and quality control

Fin clippings or muscle samples were collected and preserved in 70% ethanol for further DNA extraction and genotyping. Genotyping of the Group-SA samples was performed with a custom 46,115 SNP array (Illumina Inc., San Diego, CA 92122, USA), and genotyping of the Group-FN samples was performed with a custom 62,044 SNP array (Thermo Fisher Scientific, Waltham, MA 02451, USA). Quality control was performed using PLINK v1.09 software [30]. SNPs exhibiting deviation from Hardy‒Weinberg equilibrium (*p* < 1e^−10^), a minor allele frequency below 5% [Additional file 1, Figure S7], and a calling rate less than 80% [Additional file 1, Figure S8] were excluded. In addition, samples featuring more than 5% missing genotypes were omitted from the analysis. Following quality control, 34,492 SNPs (74.8%) and 714 animals were retained for Group-SA, whereas 48,613 SNPs (78.4%) and 707 animals were retained for Group-FN [Additional file 1, Figure S9]. All missing genotypes were imputed with Beagle v5.4 software using default parameters. After quality control, a total of 12,602 SNPs were identified as overlapping between the two molecular marker matrices, representing the genetic markers shared by Group-SA and Group-FN.

### Estimation of the genomic relationship matrix (GRM)

The genomic relationship matrix (GRM) was constructed from the genotype data using the ‘Gmatrix’ function of the AGHmatrix package in R [31], which implements the VanRaden method (method 1) as described by VanRaden [32]. Separate GRMs were estimated for each environment, as the sets of animals differed between groups—one for the seawater population $\left( {K_{{g_{SA} }} } \right)$ and another for the freshwater population $\left( {K_{{g_{FN} }} } \right)$.

### Heritability estimation

Heritability (h2) was calculated using two complementary modeling frameworks—a Bayesian approach and a generalized linear mixed model (GLMM) approach, thereby strengthening the reliability of the inference. In both seawater (Group-SA) and freshwater (Group-FN) environments, h2 was defined as, $h^{2} = \sigma_{g}^{2} /\sigma_{p}^{2}$ with variance components derived either from the Bayesian RKHS model with the software BGLR [33] or from the GLMM fitted under GMMAT software [34]. The response variable, maturity, classified fish as either 0 (immature) or 1 (precocious mature). For Group-SA, the linear predictor included the intercept, CENTER, and WFE as fixed effects, whereas for Group-FN only the intercept was considered. The genomic relationship matrix (GRM) for each group was computed using the VanRaden method (method 1) [32]. In the Bayessian approaches, genetic $(\sigma_{g}^{2} )$, residual $(\sigma_{\varepsilon }^{2} )$, and phenotypic $\left( {\sigma_{p}^{2} = \sigma_{g}^{2} + \sigma_{\varepsilon }^{2} } \right)$ variances were obtained from posterior samples across 30,000 iterations, with a burn-in of 15,000 and thinning interval 10. Posterior means and standard deviations of $h^{2}$ were calculated from iterations 1501–3000 [Additional file 1, Figure S10]. In the GLMM framework, variance components were estimated directly using the average-information REML (AI-REML) algorithm [34], providing the additive genetic and residual contributions required to compute $h^{2}$ [34].

### GWAS models

For the GWAS, a generalized linear mixed model (GLMM) was fitted using the GMMAT R package (version 1.5.0) [34]. The model assumed a Bernoulli distribution with a logit link function, where the probability of maturation for the *i-th* individual $\left( {\mu_{i} } \right)$ was modelled as a function of fixed and random effects. The glmmkin function was used to perform the GWAS by fitting the GLMM. The glmm.score function was then applied as a computationally efficient genome-wide screening test under the null hypothesis $H_{0} :\beta_{j} = 0$, and the glmm.wald function was subsequently used to estimate the additive effects ${\hat{\mathbf{\beta }}}_{{\text{j}}}$ and standard errors for SNPs that surpassed genome- or chromosome-wide significance thresholds. The complete analysis pipeline and implementation details are provided in Additional file 3.

For the seawater group (Group-SA), the generalized linear mixed model (GLMM) was defined as:

1 $$\begin{aligned} \:{\text{logit}}\left( {\mu \:_{i} } \right) = \;& \alpha _{0} + \alpha _{1} \times \:{\mathbf{CENTER}}_{i} \\ & + \alpha _{2} \times \:{\mathbf{WFE}}_{{\text{i}}} + \alpha _{3} {\mathbf{M}}_{i} + {\mathbf{b}}_{{\text{i}}} \\ \end{aligned}$$

where $$\mu_{i} = P($$$${\mathbf{MATURITY}}_{{\text{i}}} = 1$$$$|{\mathbf{CENTER}}_{i} ,$$$${\mathbf{WFE}}_{{\text{i}}} ,$$$${\mathbf{M}}_{{\text{i}}} ,$$$${ }{\mathbf{b}}_{{\text{i}}})$$ represents the expected probability of being an early-maturing male. The coefficient $${\upalpha }_{0}$$ corresponds to the intercept; $$\alpha_{1}$$ is the coefficient associated with the effect of culture center $$({\mathbf{CENTER}}_{i} ); {\upalpha }_{2}$$ is the coefficient associated with whole-fish-equivalent weight $$({\mathbf{WFE}}_{{\text{i}}} )$$, included as a covariate related to growth to reduce confounding between body weight and sexual maturation; and $$\alpha_{3}$$ is the coefficient associated with the marker effect $$({\mathbf{M}}_{i} )$$*,* representing the additive effect of the tested variant. The random effect $${\mathbf{b}}_{{\text{i}}}$$ represents the additive genetic effect of individual *i*, assumed to follow a multivariate normal distribution $${\mathbf{b}}_{{\text{i}}} \sim {\text{N}}\left( {0,{\upsigma }_{{{\text{g}}_{{{\text{SA}}}} }}^{2} K_{{{\text{g}}_{{{\text{SA}}}} }} } \right),{\text{ where }}K_{{{\text{g}}_{{{\text{SA}}}} }}$$ is the GRM and $${\upsigma }_{{{\text{g}}_{{{\text{SA}}}} }}^{2}$$ is the additive genetic variance component in Group-SA. The residual variance (dispersion parameter) was fixed at 1, following the standard convention for binomial responses in GLMMs. This specification ensures correct scaling of the logit link function while capturing the additive genetic covariance among related individuals.

For the freshwater group (Group-FN), a reduced model was fitted that included only the intercept and the marker effect as fixed effects, because information on CENTER and WFE was unavailable in this dataset. The random component remained the same as in the seawater model, representing the additive genetic effect of each individual $$({\mathbf{b}}_{{\text{i}}} )$$, which was assumed to follow a multivariate normal distribution $${\mathbf{b}}_{{\text{i}}} \sim {\text{N}}$$$$\left( {0,{\upsigma }_{{{\text{g}}_{{{\text{FN}}}} }}^{2} K_{{{\text{g}}_{{{\text{FN}}}} }} } \right)$$ , where $$K_{{{\text{g}}_{{{\text{FN}}}} }}$$ is the environment-specific genomic relationship matrix and $${\upsigma }_{{{\text{g}}_{{{\text{FN}}}} }}^{2}$$ is the additive genetic variance estimated for the freshwater population. This approach allowed the model to capture the genetic covariance among individuals while enabling accurate estimation of the additive genetic variance within the freshwater environment.

The logarithm of odds (LOD) statistic was calculated for each SNP, and significance was determined based on Bonferroni thresholds to ensure the strict control of type I error for multiple testing.

For the sea environment, thresholds were calculated for the entire genome (Eq. (2)) and per chromosome (Eq. (3)):

2 $$- \log_{10} \left( {\frac{\alpha }{tg}} \right) = 5.838748$$

3 $$- \log_{10} \left( {\frac{\alpha }{tc}} \right) = 4.376212$$

where α denotes the significance level (α = 0.05), $tg = 34,492$ represents the total number of analyzed SNPs, and $tc = 1189$ denotes the average number of SNPs per chromosome.

Similarly, for Group-FN, genome-wide and per chromosome thresholds were estimated using Eqs. (4 and 5) in accordance with [35]:

4 $$- \log_{10} \left( {\frac{\alpha }{tg}} \right) = 5.993463$$

5 $$- \log_{10} \left( {\frac{\alpha }{tc}} \right) = 4.530968$$

where α is the significance (α = 0.05), $tg = 49,253$ is the total number of SNPs in the whole genome, and $\text{tc}=1698$ is the average number of SNPs per chromosome.

For each significant SNP, the direct approach described by [9] was employed, enabling the estimation of explained variances. This estimation was computed according to Eq. (6):

6 $${\text{var}}_{{{\text{SNP}}_{{\text{j}}} }} = { }2 {\varvec{p}}_{j} {\varvec{q}}_{j} { }{{\varvec{\upbeta}}}_{{\text{j}}}^{2}$$

where $${\varvec{p}}_{j} \;{\text{and}}\;$$$${\varvec{q}}_{j} = 1$$$$- { }{\varvec{p}}_{j} { }$$ are the allele frequencies derived from the genotype data used in the association analysis, and $${{\varvec{\upbeta}}}_{{\text{j}}}$$ is the estimated additive effect for the *j*-th SNP.

The proportion of the genetic and phenotypic variance explained by the significant markers was computed as follows (Eqs. (7 and 8)):

7 $$\% {\text{var}} G_{{SNP_{j} }} = \frac{{{\text{var}}_{{SNP_{j} }} }}{{\sigma_{g}^{2} }} \times 100$$

8 $$\% {\text{var}} P_{{SNP_{j} }} = \frac{{{\text{var}}_{{SNP_{j} }} }}{{\sigma_{p}^{2} }} \times 100$$

where $\sigma_{g}^{2}$ represents the genetic variance and $\sigma_{p}^{2}$ is the phenotypic variance.

### Linkage disequilibrium (LD) analysis and haplotype construction

The LD was estimated using the pairwise correlation (r^2^) between all pairs of SNPs in the chromosomes where several significant SNPs were detected, using PLINK v1.09 software [30]. The haplotype blocks were defined according to the confidence interval (CI) algorithm proposed and implemented using Haploview v4.2 software [35]. The approach of [36] imposes limits on the CIs of the D′ value; they must be exceeded by at least 95% of all pairs [37]. Finally, the R package LocusZoom v2.1 [38] was used to visualize the regional association and LD of SNPs.

### Candidate genes

To pinpoint genes within the SNPs associated with early sexual maturation in both Group-FN and Group-SA, we utilized the annotation of version 2 of the Atlantic salmon genome (ICSASG_v2, GCF_000233375.1) using the resources provided by the NCBI Genome Data Viewer (https://www.ncbi.nlm.nih.gov/genome/gdv/?org=salmo-salar). The identification of candidate genes was performed within a region encompassing 250 kb upstream and 250 kb downstream of each significant SNP. The delineation of this range was informed by the extension and decay of linkage disequilibrium [Additional file 1, Figure S11] and the distances utilized in prior studies (3, 24, 25). If a SNP was located within two overlapping genes, both genes were analyzed as candidate genes. As supplementary information in [Additional file 2, Table S2] for Group-SA and in [Additional file 2, Table S3] for Group-FN, we updated the genomic coordinates of the significant SNPs aligned to Ssal_v3.1 using Remap, the genome remapping service tool provided by NCBI (https://www.ncbi.nlm.nih.gov/genome/tools/remap). This was done to align with the latest reference genome, enabling comparison with future studies without impacting the primary results.

## Results

### Heritability estimate

The heritability of early maturation estimated using the Bayesian RKHS model (BGLR) was high in both environments, reaching 0.79 (SD = 0.10) in seawater and 0.62 (SD = 0.10) in freshwater. Re-estimation using a GLMM approach yielded slightly lower but consistent values (0.62 and 0.54, respectively), showing a similar pattern across methods. Overall, both analytical approaches indicate higher heritability for early maturation in the seawater group compared with the freshwater group.

### GWAS in sea water

**Fig. 1:**
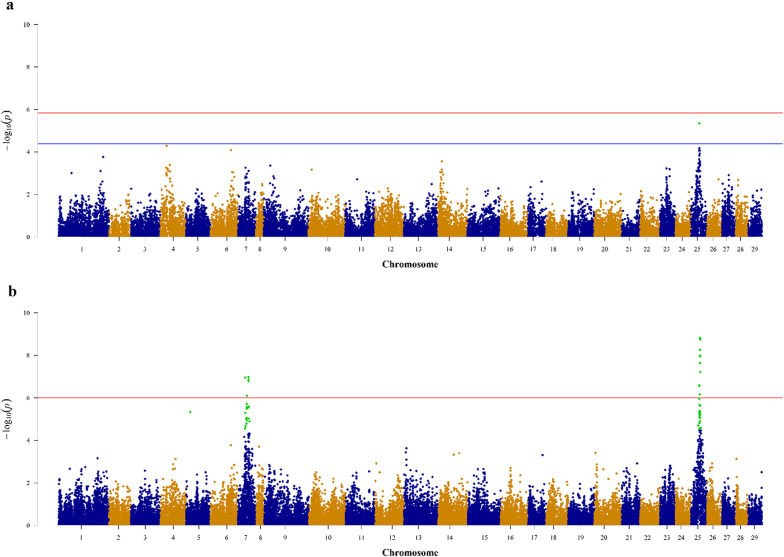
Manhattan plots of the maturation of the Lochy strain. Manhattan plots of GWAS for **a** Group-SA and **b** Group-FN. The significance thresholds at the chromosomal and genome-wide levels are represented by the blue and red lines, respectively. The green dots indicate the significant SNPs associated with sexual maturation in the Lochy strain of *Atlantic salmon*. These SNP were detected on Ssa25 for Group-SA and on Ssa5, Ssa7, and Ssa25 for Group-FN

After applying a chromosome-wide significance threshold, one SNP were associated with early sexual maturation in Group-SA (Fig. 1a) and [Additional file 1, Figure S12a]. The SNP accounted for substantial genotypic (42.9%) and phenotypic (26.6%) variance, as outlined in Table 2. One candidate gene was identified in close proximity to this significant SNP (Table 2) and [Additional file 3, Table S2]. The candidate gene was *ITPRID2* (sperm-specific antigen 2-like) gene.

**Table 2:** Top 14 out of 60 significant SNPs associated with early maturation in Group-SA and Group-FN

Gr	Tag SNP	Chr	Position	Haplo Block	Freq	*p* value	Effect	Var G%	Var P%	Harboring gene	Nearest gene
SA	25:27,034,108	25	27,034,108	–	0.58	5,932 E-06 ^(*)^	–1.20	42.9	26.6	–	Protein ITPRID2
FN	5:12,841,139	5	12,841,139	–	0.82	5.199 E-06 ^(*)^	0.87	18.9	10.2	*cystm1*-like	teneurin-2
FN	7:23,669,948	7	23,669,948	1	0.82	4.318 E-06 ^(*)^	0.92	21.2	11.5	*sh3tc1*-like	LOC106608904
FN	7:23,681,850	7	23,681,850	1	0.55	1.600 E-05 ^(*)^	0.63	16.8	9.1	Protein CABP2-like	LOC106608904
FN	7:26,530,462	7	26,530,462	2	0.73	9.496 E-06 ^(*)^	0.74	18.0	9.7	*shroom4*-like	*tnfsf10l*
FN	7:26,653,132	7	26,653,132	2	0.73	1.575 E-05 ^(*)^	0.70	16.7	9.0	*tnfsf10l*-like	LOC106609049
FN	7:27,467,174	7	27,467,174	–	0.87	3.504 E-11 ^(*; **)^	1.70	53.7	29.1	*picalm*	LOC106609053
FN	7:27,530,775	7	27,530,775	3	0.65	8.300 E-06 ^(*)^	0.69	18.3	9.9	–	rab-38-like
FN	7:33,013,514	7	33,013,514	4	0.73	1.15 E-07 ^(*; **)^	0.89	26.0	14.1	*upf2*-like	calu-A-like
FN	25:26,494,220	25	26,494,220	1	0.57	5.29 E-06 ^(*)^	–0.71	21.0	11.4	Protein ZNF285B-like	sestd1-like
FN	25:26,860,561	25	26,860,561	2	0.62	7.159 E-06 ^(*)^	–0.71	19.8	10.7	–	*itga4*
FN	25:28,623,749	25	28,623,749	3	0.58	1.34 E-05 ^(*)^	–0.67	18.4	10	*chmp2b*	*vgll3*
FN	25:28,656,101	25	28,656,101	4	0.56	1.143 E-08 ^(*; **)^	–0.90	34.0	18.4	*vgll3*	*chmp2b*
FN	25:29,697,092	25	29,697,092	5	0.53	6.126 E-08 ^(*; **)^	–0.86	31.2	16.9	LOC106586540	Protein APOA4

The *p*-value column includes (*) to indicate chromosome-wide and (**) for genome-wide significance. For Group-SA, the unique significant SNPs detected in the GWAS are shown. For Group-FN, we present the unique significant SNP detected in Ssa5, 7 significant SNPs in Ssa7 and 5 significant SNPs in Ssa25. Due to the strong linkage disequilibrium detected in some specific genomic regions in these two last chromosomes, the representative SNPs in the table were selected based on the following criteria: For Ssa7: two SNPs from haplotype block 1; two SNPs from block 2 (those with the highest and lowest estimated effects); one SNP not associated with any haplotype block but showing the strongest effect for the freshwater maturation and the SNPs with the highest effect in blocks 3 and 4. For Ssa25, the SNP with the highest significant effect within each haplotype block was selected. A total of 60 significant SNPs and their corresponding information can be found in [Additional file 3: Table S3]. Gr: Group, Tag SNP: SNP code based on chromosome and position; Chrom: Atlantic salmon chromosome; Haplo Block: Haplotype block, Freq: Allele frequency; p value: Indicates the significance of the SNP; Effect: Effect size estimate (Beta) of the effect allele 2; Var G%: Genotypic variance; Var P%: Phenotypic variance

### GWAS in freshwater

After applying chromosome-wide correction for multiple testing 60 SNPs were identified on chromosomes Ssa5 (1 SNP), Ssa7 (25 SNPs), and Ssa25 (34 SNPs), respectively. These SNPs were associated with early sexual maturation in Group-FN **(**Fig. 1b) and [Additional File 1, Figure S12b]. At the genome-wide significance threshold only 19 of those SNPs were significant. A representative subset of significant SNPs, accounting for moderate genotypic and phenotypic variances, was presented in Table 2.

**Fig. 2:**
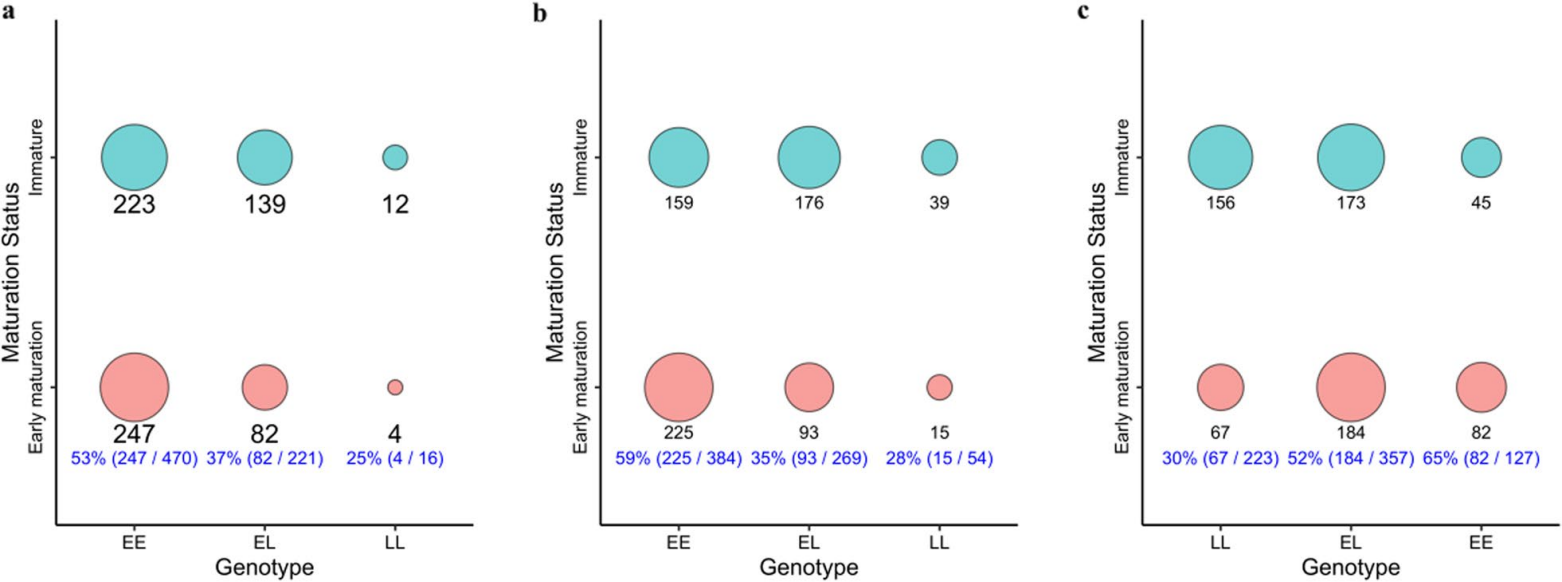
Numbers and proportions of Atlantic salmon males according to maturation status and the genotype of four SNPs. **a **Tag SNP 5:12841139 of Group-FN, harbored in the *cystm1*-like gene on Ssa5; **b** tag SNP 7:33013514 of Group-FN, harbored in the *upf2-like* gene on Ssa7; **c** tag SNP 25:28656101 of Group-FN, harbored in the *vgll3* gene on Ssa25

Some of the candidate genes identified included *cystm1-like, sh3tc1-like, shroom4-like, upf2-like, ZNF285B-like, chmp2b, vgll3* and APOA4. The description of the total of 60 significant SNPs and their corresponding information can be found in [Additional file 3, Table S3]. High linkage disequilibrium was observed between significant SNPs distributed in 4 haplotype blocks on chromosome Ssa7 [Additional File 1, Figure S13] and 5 haplotype blocks on chromosome Ssa25 [Additional File 1, Figure S14]. A total of 33 candidate genes harbored at least one SNP significantly associated with early maturation in Group-FN, one on chromosome Ssa5, 17 on chromosome Ssa7 and 15 on chromosome Ssa25 [Additional file 3, Table S3]. The genes located in proximity to the most significant SNPs on chromosomes ssa7 and ssa25 are depicted in [Additional File 1, Figure S15] and [Additional File 1, Figure S16], respectively. Furthermore, promising markers for assisted selection in this environment included the only significant SNP expressed on chromosome ssa5 (SNP 5:12841139), harbored in *cystm1*-like; SNP 7: 33013514 one of the three significant SNPs harbored in *upf2*-like gene on Ssa7; and SNP 25:28656101 harbored in gen *vgll3* on Ssa25 [Additional file 3, Table S3]. Figure. 2a, b & c shows the number and proportion of early maturing and immature fish for each SNP respectively. For the SNPs on Ssa 5 (Figs. 2a) and Ssa7 (Figs. 2b), the minor allele delayed the odds of early maturation in 0.56- and 0.44-fold, respectively. While the minor allele of one SNP in *vgll3* increased the odds of early maturation by 2.02-fold (Fig. 2c) and [Additional file 3, Table S4].

## Discussion

Genome-wide association analysis revealed 60 markers associated with early maturation in freshwater but only 1 in seawater. Low number of markers detected in seawater may be due mainly to a more unbalanced sampling of immature and early mature phenotypes in comparison with freshwater sampling (Group-SA = immature: 634; precocious maturity: 80; Group-FN = immature: 374; precocious maturity: 333). The low number of mature fish collected in seawater may be a direct consequence of their exposure to an artificial continuous light photoperiod from the time of sea stocking, which is known to inhibit the expression of the early maturation phenotype [16]. The disparity in case–control ratios, particularly in Group-SA, could increase the rate of false positives, as highlighted in studies on GWAS case–control imbalance [39], which could lead to the detection of spurious signals. However, in our study, despite the small sample size, the detected markers also might represent small or isolated effects. While the possibility of false positives cannot be entirely excluded, our findings align with previous studies that have associated our genomic signals and candidate genes with sexual maturation, as described later. Further validation with larger datasets or using methods like SAIGE [39] to address case–control imbalance could be employed to support the robustness of these associations. However, despite the imbalance between precocious and non-mature fish in seawater, similar and high levels of heritability were estimated for early maturation in the Lochy strain across both environments: 0.79 for Group-SA and 0.62 for Group-FN. As noted by [5, 21], salmonids typically exhibit a moderate to large heritable component for reproductive traits, and our estimated heritabilities to early maturation were in the range of most published reports on similar traits in domesticated populations. For example, [2] reported a high heritability of 0.90 for early male maturation, [9] reported values of 0.15 and 0.20 for freshwater and seawater maturation, respectively, and [24] reported a range of 0.54–0.84 for early maturation.

Early sexual maturation in farmed Atlantic salmon is an undesirable phenomenon that has proven challenging to eradicate. Consequently, extensive research has been conducted to develop genetic strategies to mitigate its occurrence. In this study, we provide evidence supporting that early male maturation in the Lochy strain, as in other populations, could be primarily influenced by loci of large effect, with possible contributions from additional loci of smaller effect signals. In our study, although multiple significant SNPs were identified in close proximity on Ssa25, this region could represent a single association signal. [22] highlight that a single variant may sufficiently account for the association at the ssa25 candidate region, specifically pointing to *vgll3*. Similar to findings in other European-origin populations [23–25], we identified significant SNPs associated with early maturation in the *vgll3* gene and a single association signal*.* Such evidence reinforces its central role at this locus [54]. *Vgll3* plays a role in regulating adiposity and maturity in mice [40], and [41] suggested its role in lipid regulation and maturity in juveniles of Atlantic salmon. This gene also participates in restricting pubertal testicular growth in male Atlantic salmon [42]. The regulatory role of *vgll3* has been further substantiated through evidence of cis-regulatory differences and alternative isoform expressions linked to this life history trait in *S. salar* [43]. Moreover, expression studies have shown that *vgll3* interacts with reproductive axis genes and pathways, particularly influencing gonadotropin-related gene expression as *fshb*, *lhb* in immature male Atlantic salmon [44]. This study identified other genes on Ssa25 relevant to early sexual maturation, including *APOA4*, located 25.7 kb upstream of the SNP at position 29,697,092 bp. This gene encodes the Apolipoprotein A-IV, which has been associated with heightened cholesterol levels in maturing male Atlantic salmon, potentially reflecting the increased cholesterol demand for steroidogenesis [45]. Another gene of interest is *dnajc15*, located 5.8 kb upstream of the SNP at position 28,819,973 bp [Additional file 3, Table S3], which has been associated with an inhibitory function in testicular development in mice [46].

The discovery of a strong association signal for early maturation on Ssa7 is of important interest, as we identified several SNPs located within genes previously linked to reproductive traits in Atlantic salmon and other vertebrates. The most interesting signal associated with early maturation in Ssa7 was harbored in the *Picalm* gene, a well-known vertebrate gene related to spermiation and regulation by androgens [47]. In our study the *Picalm* SNP (7:27467174) explained the most genotypic (53.7%) and phenotypic (29.1%) variances for early sexual maturation (Table 2). Recently, [9] also identified a significant association with maturation in freshwater salmon harbored in a *picalm-*like gene [4], but on chromosome 11. Although the presence of the *picalm* gene on Ssa7 has not been previously associated with maturation in Atlantic salmon, future investigations should explore its expression and functionality. Other candidates for Ssa7 include the c*opine-8* gene, which might play roles in prostate regulation and development in human males [48], the gene encoding CABP2-like gene, which is involved in rat spermatogenesis [49]; and the *tnfsf10-like* gene, whose elevated expression in the testicular tissue of human males has been suggested to induce apoptosis during spermatogenesis, potentially disrupting the process [50]. Interestingly, *tnfsf10-like* has also been related to the maturation process in male zebrafish [51]. Thus, several new candidate genes on Ssa7 appear to be associated with early maturation in Atlantic salmon.

In addition to SNPs on Ssa25 and Ssa7, we identified other candidate locus on early maturation on Ssa5. In this chromosome, a single SNP associated with early maturation was detected adjacent to *cystm1-like* and *teneurin-2* genes*.* Among those, *teneurin-2* has been associated with the transition between sexes in clown fish [52], and playing a crucial role in neuronal connection formation [53].

## Conclusions

In our GWAS of the genetic architecture of early sexual maturation in the Lochy strain of Atlantic salmon, we identified significant associations on chromosomes 5, 7, and 25 for both freshwater and sea environments, supporting the complex nature of this trait. The estimated heritability and significant SNPs associated with early maturation in the Lochy strain of Atlantic salmon valuable insights that can inform and shape effective selection strategies against early sexual maturation. Our findings present valuable insights for selective breeding programs, aiming to address the challenges posed by precocious maturation in Atlantic salmon aquaculture.

## Supplementary Information

Below is the link to the electronic supplementary material.

Supplementary Material 1.

Supplementary Material 2.

Supplementary Material 3.

## Data Availability

The phenotypic data, their analysis and the scripts for the GWASs can be downloaded from the supplementary material: phenotypic data [Additional file 2, Table S1] and phenotypic and genomics scripts [Additional File 3, R script]. The genomic data that support the findings of this study were obtained from Salmones Camanchaca Company, and restrictions apply to their availability.
