# Supplementary material for: GWAS on early sexual maturation across freshwater and seawater environments in domesticated Lochy strain of Atlantic salmon
Source: Genet Sel Evol. 2026 Jan 3;58:3. doi: 10.1186/s12711-025-01026-5 (PMC12784476; doi:10.1186/s12711-025-01026-5)
Supplement: Supplementary file 3 — Supplementary Material 3. [file 12711_2025_1026_MOESM3_ESM.docx]

***Supplementary Material***

**GWAS on early sexual maturation across freshwater and seawater environments in domesticated Lochy strain of Atlantic Salmon**

Patricia Rivera^1^**^†^**, M. Angélica Rueda-Calderón^1^**^†^**, Nicol Delgado^1^, María Eugenia López^2^, Anti Vasemägi^2,3^, Carlos Soto ^4^, Alfonso Romero^4^, José Gallardo-Matus^1*^

^1^Laboratorio de genética y genómica aplicada, Escuela de Ciencias del Mar, Pontificia Universidad Católica de Valparaíso, Chile

^2^Institute of Freshwater Research, Department of Aquatic Resources (SLU Aqua), Swedish University of Agricultural Sciences, Stångholmsvägen 2, 17893 Drottningholm, Sweden.

^3^Chair of Aquaculture, Institute of Veterinary Medicine and Animal Sciences, Estonian University of Life Sciences, Kreutzwaldi 46, 51006 Tartu, Estonia.

^4^Genetics, Reproduction and R&D Area, Salmones Camanchaca, Chile

* José Gallardo-Matus

† These authors have contributed equally to this work and share first authorship.

E-mail addresses:

PR: patricia.rivera.m@mail.pucv.cl

MARC: maria.rueda.c@pucv.cl

ND: nicol.delgado.g@mail.pucv.cl

CS: csotov@camanchaca.cl

AR: [alfonso.romero@camanchaca.cl](mailto:alfonso.romero@camanchaca.cl)

ML: [me.lopez@slu.se](mailto:me.lopez@slu.se)

AV: [anti.vasemagi@slu.se](mailto:anti.vasemagi@slu.se)

JGM: jose.gallardo@pucv.cl

**Supplementary Figures**

**
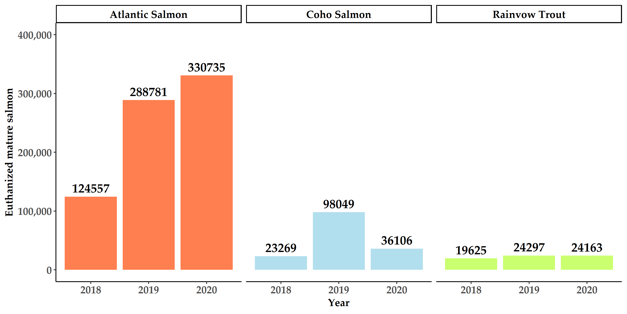
**

**Figure S1.** Statistics on euthanized Atlantic salmon and other salmonid fishes cultured in farms in Chile due to early sexual maturation (Sourse: Sernapesca).


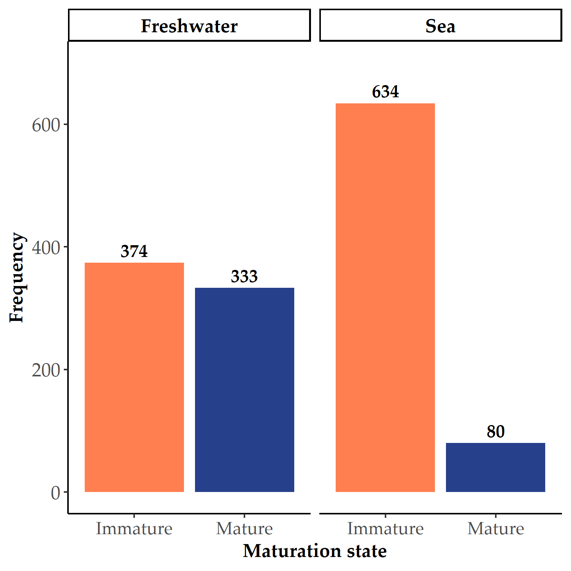


**Figure S2.** Fish collected for the early maturing GWAS study from two marine culture centers (marine environment, Group-SA) and a freshwater fish farm (Group-FN). The frequency of precocious mature fish sampled does not reflect the level of early maturity at the population level.


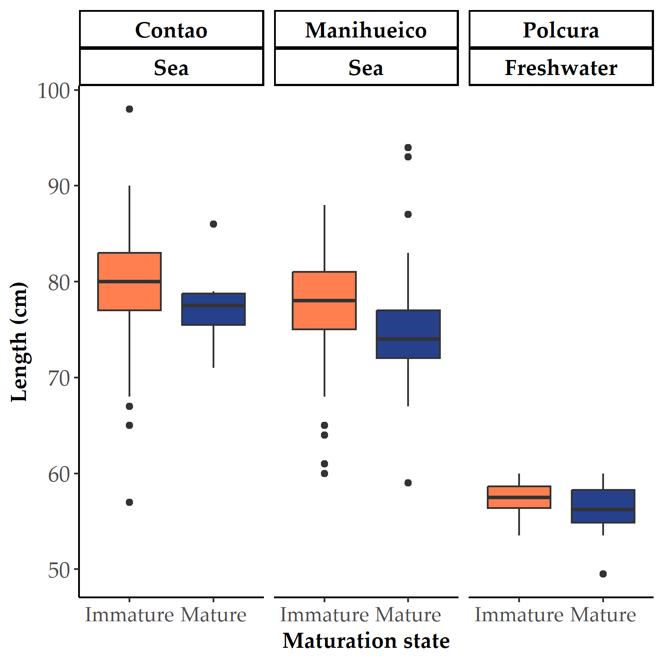


**Figure S3.** Comparison of length (cm) between precocious mature and immature males of Lochy strain of *Atlantic salmon* in two culture centers of marine environment (Group-SA) and one culture center in freshwater environment (Group-FN).


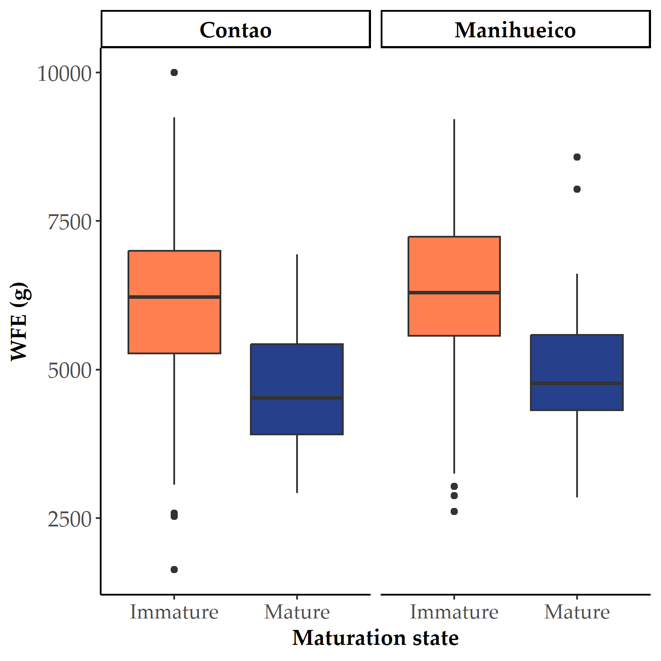


**Figure S4.** Comparison of WFE (Whole fish equivalent weight) between precocious mature and immature males of Lochy strain of *Atlantic salmon* in the two culture centers of the sea.

**
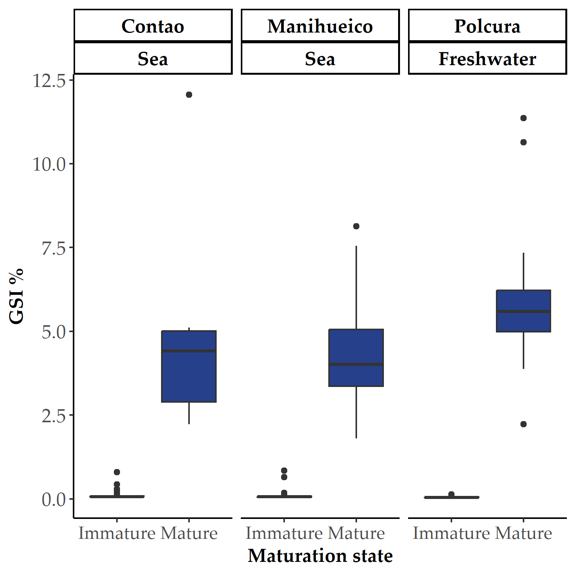
**

**Figure S5.** GSI comparison between males of Lochy Strain of Atlantic salmon by environment and maturation state.

**
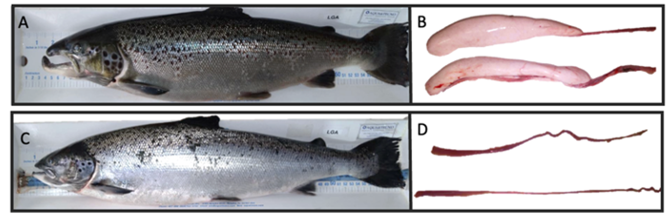
**

**Figure S6.** Comparison between maturation stages in males of Lochy strain of *Atlantic salmon*. A) Early mature male; B) Gonad of early mature male; C) Immature male; D) Gonad of immature male.

**
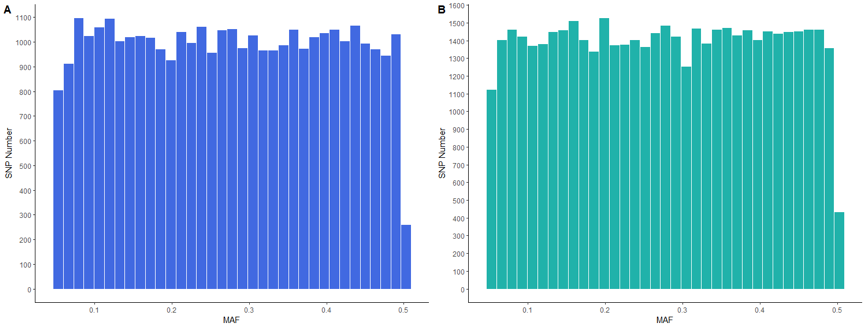
**

**Figure S7.** MAF Minor allele frequency. A) Group-SA (Marine environment-2021), B) Group-FN (Freshwater environment – 2022).


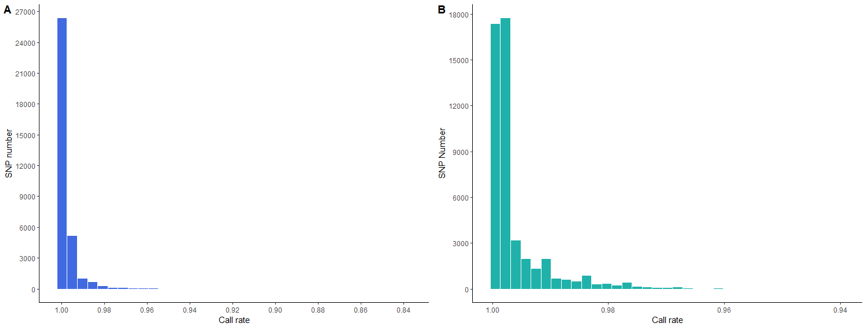


**Figure S8.** Variant call rate before imputing missing genotypes with Beagle v5.4. A) Group-SA (sea environment-2021), B) Group-FN (Freshwater environment – 2022).

**
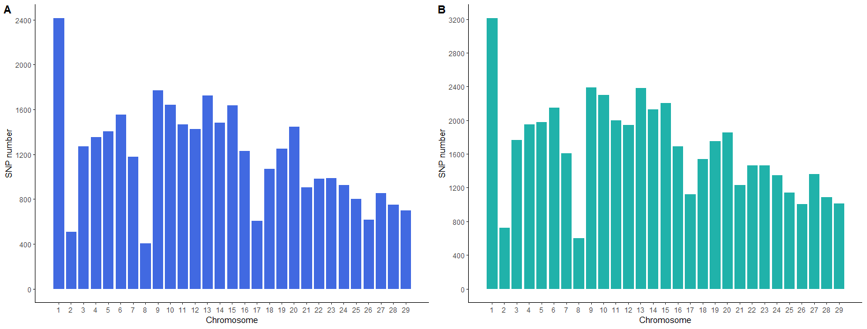
**

**Figure S9.** Number of SNPs per chromosome after quality control. A) Group-SA (Marine environment-2021), B) Group-FN (Freshwater environment – 2022).

**
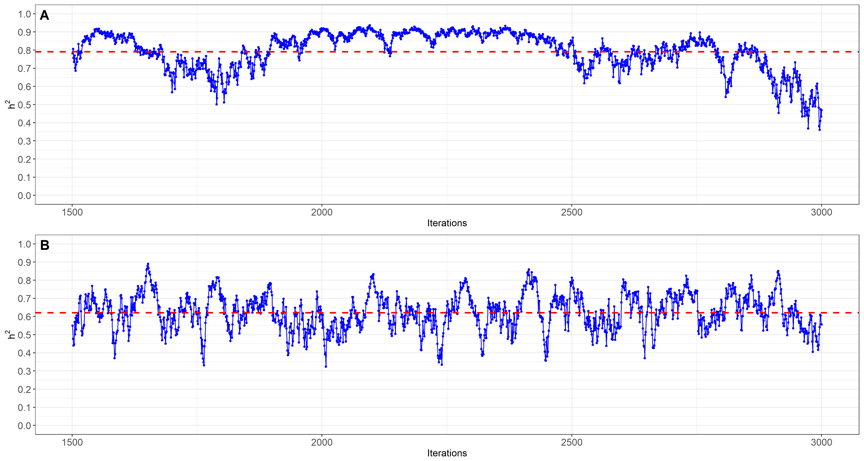
**

**Figure S10.** A). Plot of heritability ($h^{2}$) estimates across iterations for Group-SA (seawater environment). B). Plot of heritability ($h^{2}$) estimates across iterations for Group-FN (freshwater environment). The blue line represents the observed values, and the red dashed line indicates the final heritability estimate ($h^{2}$). Heritability ($h^{2}$) was calculated as $h^{2}= {\sigma_{g}^{2}}/{\sigma_{p}^{2}}$ , where $\sigma_{g}^{2}$​ represents the genetic variance and $\sigma_{p}^{2}$​ the phenotypic variance. Estimates were based on 30,000 iterations of the Bayesian Generalized Linear Regression (BGLR) model, with a burn-in of 15,000 and a thinning interval of 10. The mean and standard deviation of $h^{2}$ were calculated from the posterior distribution of iterations 1,501 to 3,000.


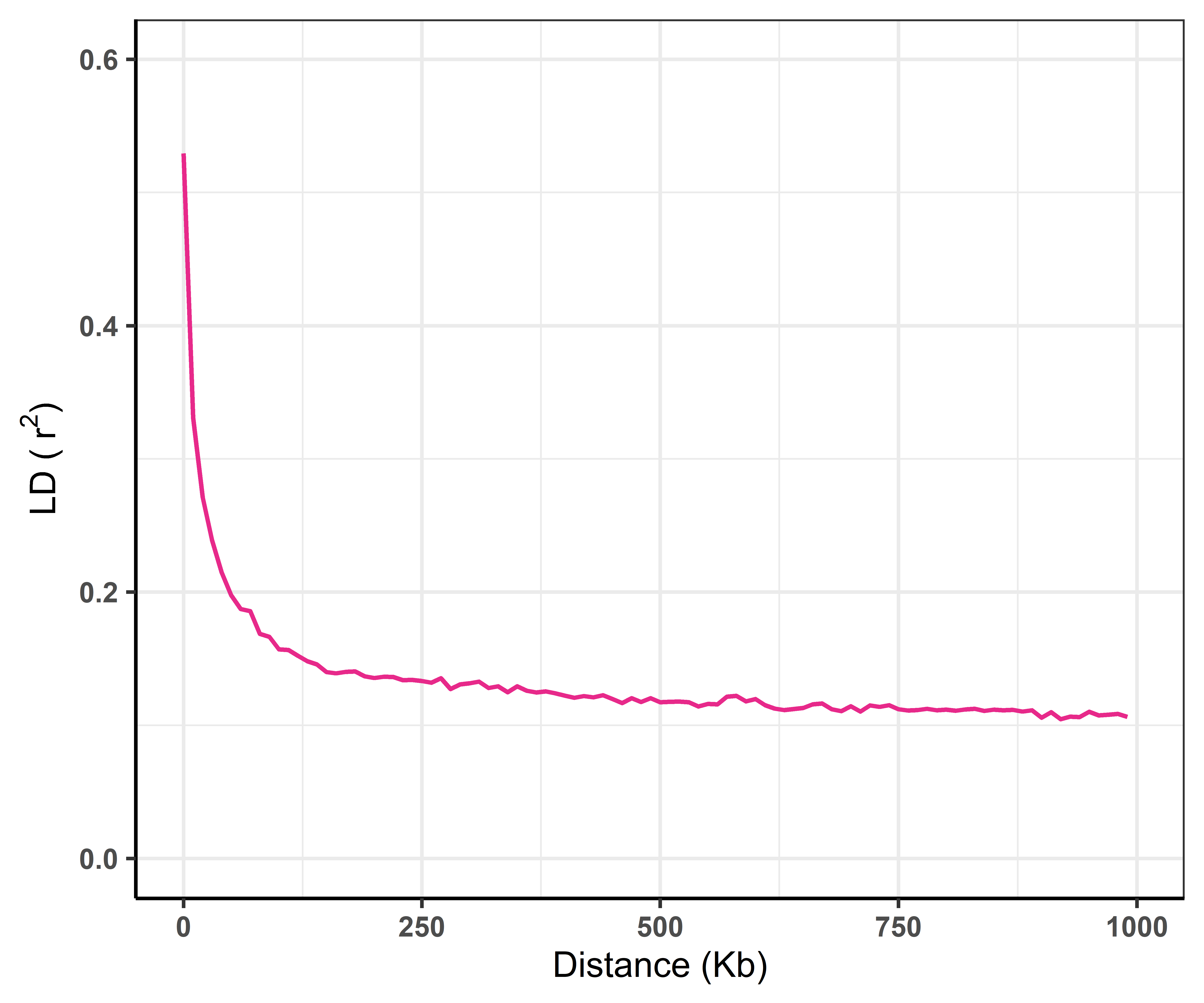


**Figure S11.** Extent and decay of linkage disequilibrium (LD) with distance.


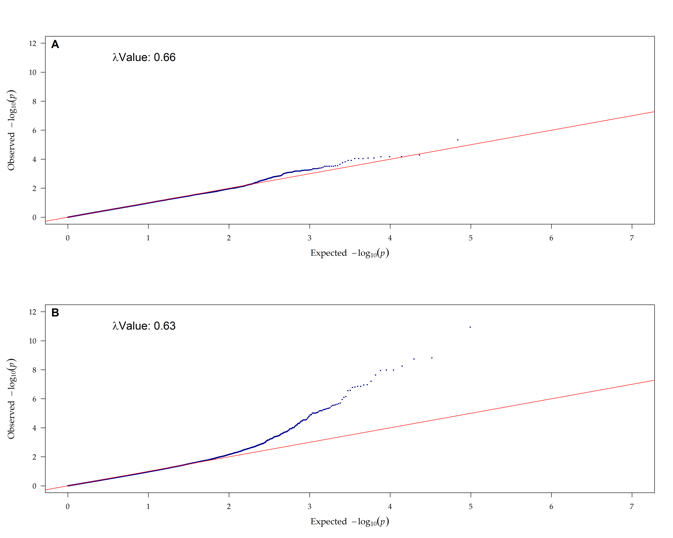


**Figure S12.** A). Quantile-quantile plot of GWAS results for early sexual maturation in Group-SA (seawater environment). B). Quantile-quantile plot of GWAS results for early sexual maturation in Group-FN (freshwater environment). The plots compare the distribution of observed versus expected p-values, showing no evidence of general p-value inflation.


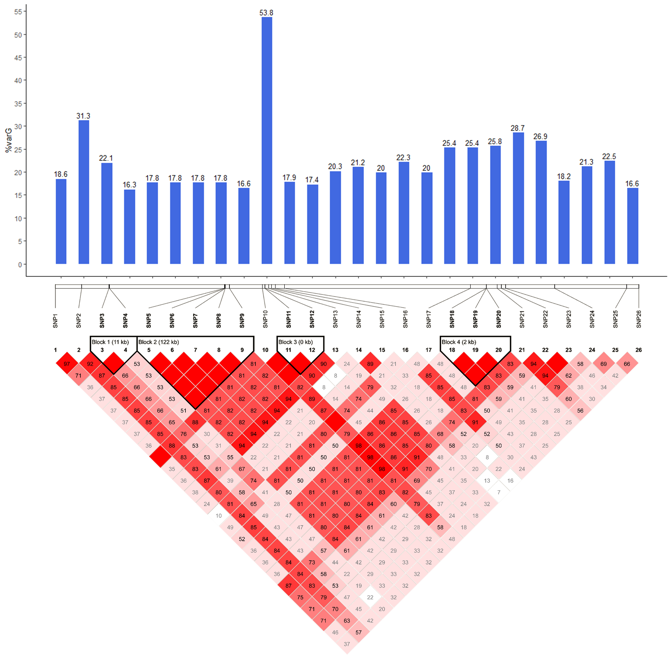


**Figure S13.** The haplotype blocks and pairwise linkage disequilibrium values (D’) for the SNPs in chromosome 7 for GWAS of the Group-FN, with the genetic variance explained by each significant QTL detected. The values within boxes are pair wise SNP correlation (D’), bright red boxes without numbers indicate complete LD (D’ = 1). The brighter shade of red indicates higher linkage disequilibrium.

**
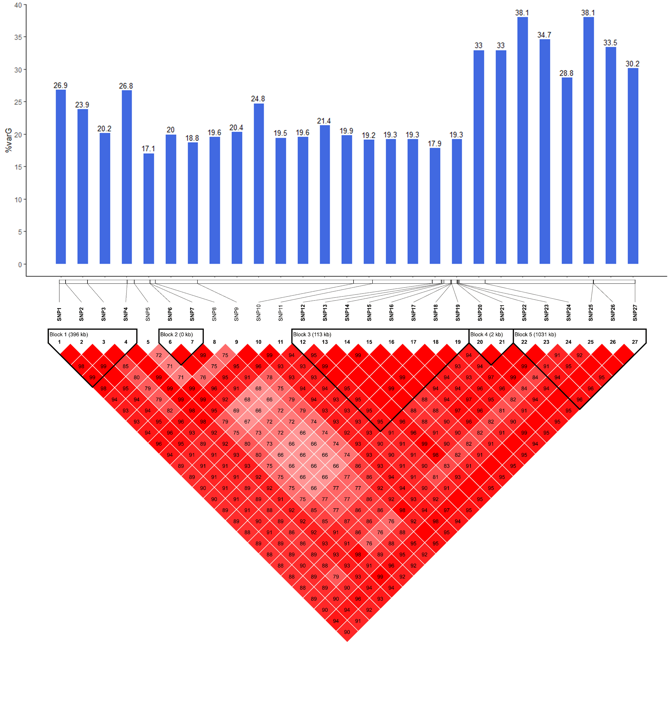
Figure S14.** The haplotype blocks and pairwise linkage disequilibrium values (D’) for the SNPs in chromosome 25 for GWAS of the Group-FN, with the genetic variance explained by each significant QTL detected**.** The values within boxes are pair wise SNP correlation (D’), bright red boxes without numbers indicate complete LD (D’ = 1). The brighter shade of red indicates higher linkage disequilibrium.


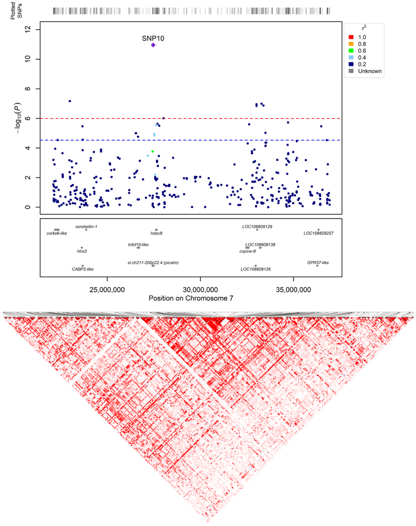


**Figure S15. LocusZoom and linkage disequilibrium plot of significantly associated genetic region in chromosome 7 to GWAS for early sexual maturation trait in Group-FN:** Plot of the genomic region showing genome-wide significant association to freshwater early sexual maturation trait [Additional file 3: Table S4]. GWAS result (on a –log10 P scale; left y axis) is shown for nearby SNPs to the index SNP (that is, the SNP with the smallest P value, purple diamond). In Ssa7 the strongest significance was to SNP10. The marker (dot) color indicates the strength of LD (r2) between the index SNP and the SNPs on chromosome 7. Genes in this region and genomic coordinates (in pb, using the NCBI Atlantic salmon genome sequence, [ICSASG_v2](https://www.ncbi.nlm.nih.gov/datasets/genome/GCF_000233375.1/), as reference) are shown at the bottom. Plot was produced with the LocusZoom R package. Below this region is also shows an LD heatmap (using D’, ranging from red indicating D’=1 to white indicating D’=0) produced using Haploview.


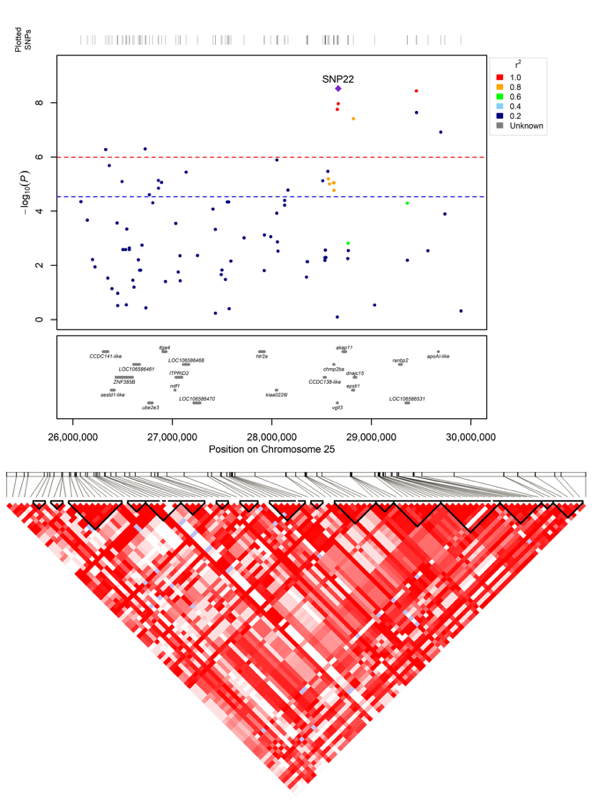


**Figure S16. LocusZoom and linkage disequilibrium plot of significantly associated genetic region in chromosome 25 for early sexual maturation trait GWAS in Group-FN:** Plot of the genomic region showing genome-wide significant association to freshwater early sexual maturation traits [Additional file 3: Table S4]. GWAS result (on a –log10 P scale; left y axis) is shown for nearby SNPs to the index SNP (that is, the SNP with the smallest P value, purple diamond). In Ssa25 the strongest significance was to SNP22. The marker (dot) color indicating the strength of LD (r2) between the index SNP and the SNPs on Ssa25. Genes in this region and genomic coordinates (in pb, using the NCBI Atlantic salmon genome sequence, [ICSASG_v2](https://www.ncbi.nlm.nih.gov/datasets/genome/GCF_000233375.1/), as reference) are shown at the bottom. Plot was produced with the LocusZoom R package. Below this region is also shows an LD heatmap (using D’, ranging from red indicating D’=1 to white indicating D’=0) produced using Haploview.
